# Repetitive Electroacupuncture Attenuates Cold-Induced Hypertension through Enkephalin in the Rostral Ventral Lateral Medulla

**DOI:** 10.1038/srep35791

**Published:** 2016-10-24

**Authors:** Min Li, Stephanie C. Tjen-A-Looi, Zhi-Ling Guo, John C. Longhurst

**Affiliations:** 1 Department of Medicine and Susan Samueli Center of Integrative Medicine, School of Medicine, University of California, Irvine, CA 92697-4075, USA.

## Abstract

Acupuncture lowers blood pressure (BP) in hypertension, but mechanisms underlying its action are unclear. To simulate clinical studies, we performed electroacupuncture (EA) in unanesthetized rats with cold-induced hypertension (CIH) induced by six weeks of cold exposure (6 °C). EA (0.1 – 0.4 mA, 2 Hz) was applied at ST36-37 acupoints overlying the deep peroneal nerve for 30 min twice weekly for five weeks while sham-EA was conducted with the same procedures as EA except for no electrical stimulation. Elevated BP was reduced after six sessions of EA treatment and remained low 72 hrs after EA in 18 CIH rats, but not in sham-EA (n = 12) and untreated (n = 6) CIH ones. The mRNA level of preproenkephalin in the rostral ventrolateral medulla (rVLM) 72 hr after EA was increased (n = 9), compared to the sham-EA (n = 6), untreated CIH rats (n = 6) and normotensive control animals (n = 6). Microinjection of ICI 174,864, a δ-opioid receptor antagonist, into the rVLM of EA-treated CIH rats partially reversed EA’s effect on elevated BP (n = 4). Stimulation of rVLM of CIH rats treated with sham-EA using a δ-opioid agonist, DADLE, decreased BP (n = 6). These data suggest that increased enkephalin in the rVLM induced by repetitive EA contributes to BP lowering action of EA.

Approximately one in three adults has high blood pressure (BP) worldwide. Hypertension and its consequences, such as stroke and heart attacks, are enormous public health problems^1^. The 7^th^ *Joint National Committee on Prevention, Detection, Evaluation and Treatment of High Blood Pressure* report recommends lifestyle modification (i.e., non-traditional approaches) with or without pharmacological intervention^2^. Although the effect of acupuncture on hypertension is controversial^3^,^4^,^5^,^6^,^7^, acupuncture may offer a non-pharmacological approach to reduce high BP.

We have shown in previous studies of patients with mild to moderate hypertension that electroacupuncture (EA) applied once weekly over an eight-week course of therapy lowers peak systolic blood pressures (SBP) by 10 – 12 mmHg as measured by 24 hr ambulatory monitoring^7^. This response appears to persist for an additional four weeks after EA is terminated. In concert with this observed prolonged action of acupuncture, several research groups have shown significant decreases in the intensity of chronic low back and shoulder pain is maintained for one to six months after completion of acupuncture treatment provided once weekly for periods ranging between six and eight weeks, compared to placebo group^8^,^9^. Thus, acupuncture appears to reduce chronic hypertension and pain for prolonged periods of time. However, the mechanisms underlying these long-term actions are unclear.

Epidemiological data have shown that in cold regions and during cooler seasons the prevalence of hypertension and deaths from stroke and heart attack associated with hypertension are elevated^10^,^11^,^12^. A number of studies have demonstrated that rats exposed to mild cold (6 °C) for several weeks develop hypertension (cold-induced hypertension, CIH) characteristically similar to humans with essential hypertension^13^,^14^. The CIH experimental model is a “naturally-occurring” form of hypertension, associated with chronic cold-induced stress that is a common cause for essential hypertension^15^. Cold-induced hypertension is as or more clinically relevant than elevation of BP in other hypertensive models, including spontaneous hypertensive rats (SHR), DOCA-salt and renal hypertensive rats^13^,^16^, which commonly require surgical intervention, administration of excessive doses of drugs or hormones, or genetic manipulation. Importantly, sympathetic activity is increased in CIH^14^. Sympathetic outflow and ultimately BP are regulated through activation of sympathoexcitatory premotor neurons in the rostral ventrolateral medulla (rVLM)^17^,^18^. Acupuncture reduces sympathoexcitatory reflex-induced elevations in BP through modulation of presympathetic rVLM neuronal activity to, in turn, decrease sympathetic activity^19^,^20^,^21^. Thus, to simulate clinically relevant hypertension due in part to sympathoexcitation, the conscious CIH rat model was used in the present study to investigate the effect of repeated EA on BP and the mechanisms underlying EA’s action in a relevant brain stem region, the rVLM.

The opioid system in the rVLM contributes to the EA-modulatory actions on reflex hypertension in anesthetized animals^19^,^20^,^21^. Despite strong evidence showing that acupuncture influences short-term hypertensive reflexes, it is uncertain if opioids in the rVLM participate in the BP lowering actions of repetitive EA in chronic hypertension. As such, in the present study, the clinically relevant CIH model was used to examine: 1) the ability of repetitive EA to reduce sustained elevations in BP; 2) central mechanisms underlying acupuncture’s hypotensive actions (Fig. 1). We hypothesized that repetitive EA lowers CIH in conscious animals through the opioid system in the rVLM.

## Results

### Effect of repetitive EA treatment on cold-induced hypertension

Cold exposure increased both systolic and diastolic blood pressure (SBP, DBP) within four weeks and induced sustained hypertension by six weeks (Fig. 2, panels A and B). Blood pressures of rats in the untreated hypertensive and sham-EA groups remained elevated at the end of 11 weeks. In contrast, EA reduced both the elevated SBP and DBP after six sessions; the BPs remained low throughout the period of treatment relative to the sham treated group. Although SBP and DBP of EA-treated CIH rats post EA were higher than those observed immediately after termination of EA, they were still reduced three days post EA relative to the sham-EA control (Fig. 2, panels A and B). The BP of the normotensive rats remained stable throughout the 11 week period. Cold exposure increased heart rate (HR) within four weeks and remained unchanged in the untreated hypertensive, sham-EA and EA groups throughout the remainder of the 11 week period and for three days afterwards (Fig. 2C).

### Preproenkephalin mRNA expression 72 hours after EA

The rVLM preproenkephalin mRNA level was elevated 72 hr following application of 11 sessions of EA treatment in the hypertensive animals (Fig. 3). Thus, the preproenkephalin mRNA was significantly higher 72 hour after EA (14.3 ± 1) compared to the sham-EA (4.3 ± 0.5), untreated hypertensive (4.3 ± 0.6) and normotensive rats (3.8 ± 0.9).

### Opioid receptor antagonism in EA-treated CIH rats

In a preliminary study we observed that bilateral microinjection of 1 mM naloxone (50 nl; a non-specific opioid receptor antagonist) into the rVLM increased SBP, DBP and mean blood pressure (MBP) in two EA-treated hypertensive rats (140 to 146, 77 to 84, and 98 to 105 mmHg in one rat, and 132 to 144, 63 to 70, and 90 to 97 mmHg in a second rat). Heart rate was not influenced by naloxone in either rat (447 to 451, and 465 to 467 bpm, respectively). To investigate the role of the δ-opioid receptor in the EA hypotensive effect in animals with sustained hypertension, we employed specific δ-opioid receptor antagonism with ICI 174,864 (1 mM in 50 nl). We noted a slight increase in BP(~10 mmHg; Fig. 4A), but not heart rate after the initial unilateral injection. Bilateral blockade of δ-opioid receptors in the rVLM partially reversed EA’s action on systolic, mean and diastolic BPs in four additional animals (Fig. 4). Heart rates were not influenced by ICI 174,864. The onset and duration of the responses following the initial unilateral injection were 10 s and 70 min, respectively, with a peak effect observed at 3 min (Fig. 4A). Microinjection of the same volume of vehicles for naloxone and ICI 174,864 in EA treated hypertensive rats did not influence BP or heart rate (n = 4).

### Delta-opioid receptor stimulation in sham-EA CIH rats

The δ-opioid receptor agonist, DADLE (10 nM in 50 nl), microinjected bilaterally into the rVLM of six sham-EA CIH rats decreased SBP, but did not alter DBP, MBP (Fig. 5) or heart rate. We noted only a small reduction in SBP (~5 mmHg) after unilateral injection. The onset (after initial unilateral injection) and duration of the responses to DADLE were 15 and 300 s, while the peak effect was observed at 140 s. Microinjection of same volume of normal saline into the rVLM did not influence BP or heart rate in four of these six rats.

### Delta-opioid receptor stimulation and blockade in normotensive rats

Bilateral microinjection of ICI 174,864 (1 mM in 50 nl) into the rVLM of three normotensive rats did not change BP. The recordings of SBP, DBP and MBP were the same before and after the microinjection of ICI 174,864 into the rVLM of the normotensive rats, noted as 109 ± 8, 65 ± 4 and 80 ± 4 mmHg. Three bilateral microinjection of DADLE (10 nM in 50 nl) into the rVLM of the normotensive rats also did not influence BP. SBP, DBP and MBP were the same before and after microinjection of DADLE in the normotensive rats (143 ± 9, 79 ± 5 and 100 ± 6 mmHg). Heart rate was not affected by either δ-opioid receptor stimulation or antagonism.

### Microinjection sites

Examination of the rat brain slices verified that each of the injections was located within the rVLM, with the exception of three ICI 174,864 injections that were found to be outside the rVLM of EA-treated hypertensive rats. None of the injections outside the rVLM altered blood pressure. Thus, microinjection sites observed to be 1.0 to 1.5 mm rostral to the obex, 0.5 to 1.0 mm from the ventral surface and 1.8 to 2.3 mm lateral to the midline, were considered to be within the rVLM, as noted in the atlas of Paxinos and Watson^22^ (Fig. 5).

## Discussion

We have shown in a number of studies that EA inhibits sympathoexcitatory cardiovascular responses^19^,^20^,^21^,^23^. Preproenkephalin in the rVLM is increased following repetitive stimulation of the P5-6 acupoints and the underlying median nerves of normotensive rats^24^. However, we know little about mechanisms underlying EA’s influence in sustained hypertension. As such, we established a hypertensive model known to increase BP through its sympathoexcitation, to investigate the influence of EA in sustained hypertensive rats and to determine if opioids in the brain stem underlie EA’s actions. The major new findings of this study are: (1) Repetitive EA reduces sustained hypertension, (2) Reduced BP in EA-treated rats persists for at least additional three days after end of EA treatment, providing evidence for a long-lasting hemodynamic action of EA, (3) EA increases the enkephalin gene expression in the rVLM for at least three days after eleven applications and (4) Blockade of δ-opioid receptors using ICI 174,864 or naloxone in the rVLM in EA-treated hypertensive rats partly reverses the EA response while stimulation of δ-opioid receptors using exogenous DADLE in the rVLM lowers BP in sham-EA hypertensive rats, directionally mimicking the EA effect in CIH. These data suggest that the δ-opioid system in the rVLM participates in the hypotensive action of EA in rats with sustained hypertension.

The sympathetic nervous system is essential for maintaining body temperature of cold-exposed rats^15^. The responses of intact rats to cold exposure (4 °C) include vasoconstriction, piloerection, shivering, adrenocorticotropin (ACTH) hypersecretion and increased mobilization of free fatty acids and glucose^15^. Chemical sympathectomy blocks all of these responses except for ACTH hypersecretion^15^. Animals subjected to chemical sympathectomy lose body heat rapidly and die in a few hours during cold exposure. However, the damaging actions of the chemical sympathectomy are reversed by administration of catecholamines. These studies suggest that increased sympathetic outflow contributes to development and maintenance of CIH^13^,^14^. Norepinephrine in plasma increases after 24 hr of exposure to cold and remains elevated throughout the experiment, whereas the concentration of epinephrine in plasma increases early on but returns to control level after 19 days of exposure to cold^14^. We also observed that BP remained elevated even when animals were placed in a neutral environment at room temperature for several hours during application of EA and experimental procedures. Compared to the other forms of hypertension, such as spontaneous hypertension^25^, DOCA salt hypertension^26^ and renal hypertension^27^, CIH is presently the only “naturally-occurring” form of experimentally-induced hypertension that can be induced without surgical intervention, administration of excessive doses of drugs or hormones or genetic manipulation^16^. Also, as noted above, this form of hypertension has a large sympathoexcitatory component. As shown in our study and by others^13^,^28^, systolic, diastolic and mean BPs increase significantly after four weeks of exposure to mild cold (6 °C) and are sustained at an elevated level for at least another six weeks in cold environments. Importantly, BP elevation is also observed in human subjects living in cold regions and during cold seasons^10^,^29^. Considering that EA inhibits sympathoexcitatory responses^19^,^20^,^23^, we predicted that EA would reduce the chronically elevated sympathoexcitatory state in the CIH model. Acupuncture does not influence thermosensation independently, i.e., more than controls^30^,^31^,^32^ although it may raise the pain threshold^32^, including thermal pain caused by excessive warm or cold^33^,^34^. Since mild cold (6 °C) used in the present study was much less intense than the excessive cold that causes pain, it is unlikely that our observations of EA’s action on BP were due to changes in cold threshold.

Stimulation of the ST36-37 (Zusanli-Shangjuxu) acupoints overlying the deep peroneal nerves has been widely used in treating several clinical conditions, including pain, spinal cord injury, nausea, gastrointestinal diseases, allergic rhinitis and cardiovascular conditions, such as hypertension^7^,^35^. We have shown that EA at ST36-37 or P5-6 similarly inhibit sympathoexcitatory reflex responses induced by gastric distension^36^ and gall bladder stimulation^37^. Both P5-6, overlying the median nerves, and ST36-37, overlying the deep peroneal nerves, inhibit sympathoexcitatory responses for prolonged periods of time and with similar intensities^36^,^37^. Although EA at ST36-37 and P5-6 appears to ameliorate BP in patients with mild to moderate hypertension for at least four weeks after terminating treatment^7^, the underlying mechanisms are unknown. We chose ST36-37 rather than P5-6 stimulation, since this approach was technically easier in conscious animals.

Our previous clinical study demonstrated that weekly EA therapy reduces elevated SBP and DBP, but not HR in patients with mild to moderate essential hypertension, and that EA had a stronger effect on elevations in SBP than DBP^7^. The efficacy of EA in lowering SBP may be important for patients with advancing age (>60 years)^38^ since stress-related spikes in SBP places patients with hypertension at risk for stroke, developing peripheral artery disease, heart failure, as well as myocardial infarction and other cardiovascular diseases^38^. Thus, reduction in BP with EA has the potential to reduce the risk for these cardiovascular disorders. In the present study, we likewise found that EA reduced both SBP and DBP in animals with cold-induced hypertension. The present experimental study provides possible mechanisms by which EA reduces BP in patients with hypertension.

The rVLM serves an important role in cardiovascular sympathoexcitatory regulation^17^,^18^ and is an important site of EA’s action in suppressing sympathoexcitation^6^. In this regard, we have shown that the opioid system in the rVLM including endorphin and enkephalins, that largely act through stimulation of μ- and δ-opioid receptors, respectively, contributes importantly to the sympathoinhibitory effects of EA on pressor reflex^19^. Anatomical studies have documented that enkephalins but not endorphin is synthesized in EA-activated neurons in the rVLM^39^,^40^. Endorphins are synthesized in the arcuate nucleus of the hypothalamus and are transported to the rVLM through long projections^39^,^40^.

Acupuncture’s long-lasting modulation of cardiovascular excitatory reflexes is mediated, in part, by opioids in the rVLM. In this regard, one 30 min session of EA in unanesthetized animals inhibits elevated BP for four or more hrs^41^. Under anesthesia, the inhibitory effect of 30 min of EA on premotor sympathoexcitatory cardiovascular rVLM neuronal responses lasts 60-90 min^19^,^20^,^21^. In the rVLM, opioids and GABA, but not nociceptin, participate in EA-related prolonged inhibition of sympathoexcitatory cardiovascular responses^23^. Furthermore, mRNA expression of preproenkephalin in the rVLM is increased at 90 min after 30 min of EA and 24 hr after two repetitive applications of EA in anesthetized^42^ and conscious normotensive rats^24^, suggesting that molecular changes of enkephalin gene expression in the rVLM may contribute to EA’s immediate actions. Despite these prior observations, it has been unclear if increases in preproenkephalin in the rVLM in response to repetitive EA over a five week period contribute to the BP long-lasting lowering effect of EA in rats with sustained hypertension. In this regard, the current study is the first to demonstrate that repeated application of EA reduces sustained hypertension induced by cold stress for at least three days after the course of EA treatment. This prolonged action is associated with elevated preproenkephalin mRNA expression levels in the rVLM. The observation that preproenkephalin is increased in the rVLM after repeated EA in the hypertensive rats, suggests that EA-associated increases in expression of preproenkephalin enhances the availability of rVLM enkephalin to, in turn, decrease elevated neuronal activity, sympathetic outflow and ultimately hypertension. Our previous study documents that the protein (met-enkephalin) rises in parallel with its mRNA precursor, preproenkephalin, following repetitive EA^24^. These data in aggregate therefore suggest that molecular changes of enkephalin gene expression are related to EA’s long-lasting actions on elevated BP in hypertensive models.

Microinjection of a δ-opioid receptor agonist into the rVLM, like EA, decreased elevated BP in CIH rats, while ICI 174,864, the δ-opioid receptor antagonist partly reversed the EA effect. Thus, increased opioid synthesis in the rVLM associated with EA participates in the long-lasting inhibitory action of EA in CIH.

Microinjection of ICI 174,864, the δ opioid receptor antagonist, or DADLE, the δ-opioid agonist, into the rVLM of normotensive rats did not influence blood pressure. This result is consistent with our and other previous studies and indicates that there is no tonic action resulting from δ-opioid receptor stimulation^19^,^20^,^23^,^24^,^43^,^44^,^45^. Thus it appears that under normal physiological conditions endogenous opioids in the rVLM do not participate in BP regulation. Furthermore, endogenous opioid release induced by EA^24^,^39^,^40^,^42^ does not influence baseline BP. It is likely that in normotensive animals, sympathoexcitatory cardiovascular neurons in rVLM maintain a low level of activation and thus there is little for exogenous opioids to act upon. On the other hand, when rVLM neurons are activated during reflex responses^19^,^20^,^23^,^24^,^43^,^44^ or in the setting of hypertension^45^, exogenous opioids administered through microinjection or increased endogenous opioids in the rVLM in response to EA decrease pressor reflex responses and attenuate hypertension^46^,^47^. As such, increased expression of opioid receptors during the development of hypertension also contributes to an enhanced opioid system in the rVLM.

Alpha-chloralose frequently is used in cardiovascular experimentation^19^,^23^,^43^,^44^,^48^ since it preserves cardiovascular reflex function better than other anesthetics like barbiturates^49^,^50^. The actions of enkephalin on BP under α-chloralose anesthesia are similar to those observed in conscious preparations^51^. Under urethane anesthesia, 100 nl of 0.2 mM endomorphin-2 microinjected into the rVLM of normotensive Wistar rats has been shown to decrease BP and heart rate^52^. Also, in α-chloralose and urethane anesthetized rats^46^, microinjection of 100 nl of 3 nM D-Ala^2^, N-Me-Phe^4^, Gly^5^-ol]-Enkephalin acetate salt (DAGO), a μ-opioid agonist or 10 nM naloxone into the rVLM of normotensive Sprague Dawley rats respectively decreases or increases arterial pressure and heart rate. The reasons for these discrepant observations are uncertain but might be related to the different anesthetics and/or microinjection volume.

Hypertension affects about one third of the adult population of the world. It is also the leading chronic risk factor for mortality^1^. Acupuncture potentially presents a viable non-pharmacological treatment option. However, due to limitations of the previous clinical studies, including small sample sizes, methodological and design heterogeneity, and differences in BP related end-points, the actions of acupuncture in hypertension remains controversial^5^. Using a clinically relevant model of hypertension, the current study demonstrates that repetitive EA attenuates hypertension by about 40% through a δ-opioid mechanism, occurring at least in part in the rVLM. Repetitive EA evokes a long-lasting response, suggesting that this therapy may be suitable for treating clinical hypertension.

In conclusion, repetitive EA attenuates CIH. The BP lowering action of EA lasts at least three days after cessation of EA. The inhibitory effect of EA is related to the increases in enkephalin levels in the rVLM. The present study suggests that acupuncture may be a viable non-pharmacological option to treat chronic hypertension.

## Methods

**Figure 1:**
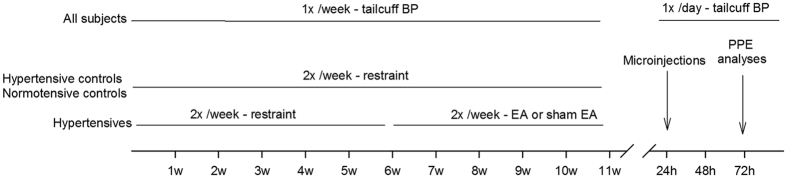
Timeline of experiments for four groups of rats. Cold-induced hypertension (CIH) rats were randomly divided into EA, sham-EA or untreated hypertension groups. They were treated with repetitive EA or sham-EA at the ST36-37 acupoints, or restraint only, for 30 min twice weekly for another five weeks following staying in the cold room for six weeks. Rats kept at room temperature were restrained for 30 min twice weekly for eleven weeks. Blood pressures (BPs) in all groups of rats were evaluated weekly using tail-cuff. Twenty-four or 72 hrs after five weeks of treatment, rats were decapitated or microinjected to respectively examine the preproenkephalin mRNA levels and role of δ-opioid receptors in the rVLM.

**Figure 2:**
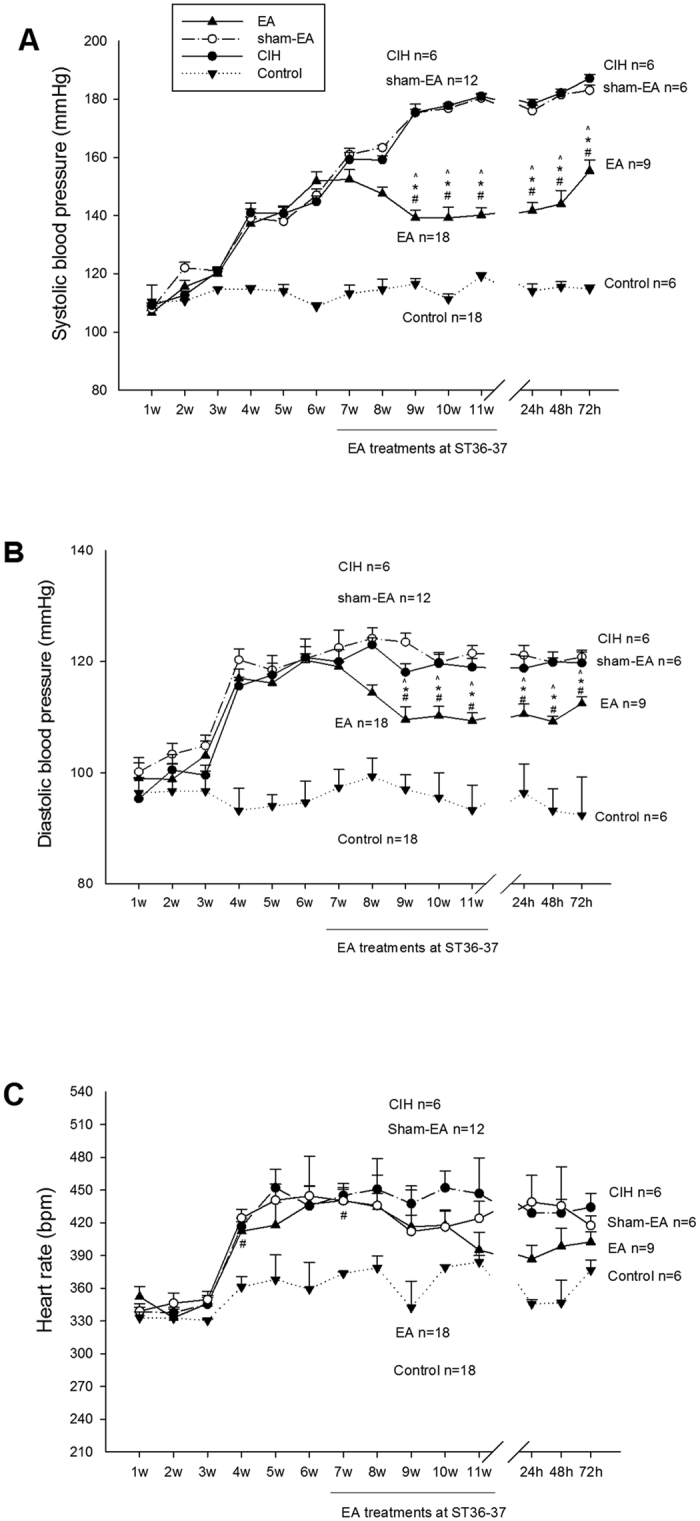
Repetitive EA treatment in cold-induced hypertension (CIH). Cold exposure increased systolic blood pressure (SBP; Panel A), diastolic blood pressure (DBP; Panel B) and heart rate (HR; Panel C) after four weeks and induced sustained hypertension by six weeks. BPs and HRs of rats in the hypertension and sham-EA groups remained elevated at 11 weeks. In contrast, elevated SBP and DBP, but not HR in the EA group was reduced after six sessions of EA and remained low throughout EA treatment. BPs and HR of normotensive rats remained stable. SBP and DBP of EA-treated CIH rats, although beginning to return to pre-treatment levels were still significantly reduced for three days following termination of EA compared to sham-EA. Note that the numbers of sham-EA, EA and normotensive rats respectively were reduced to 6, 9, and 6 rats at 72 h after termination of treatments. Values represent means ± SEM. ^, * and # indicate P < 0.05 compared with sham-EA, CIH and normotensive controls, respectively.

**Figure 3:**
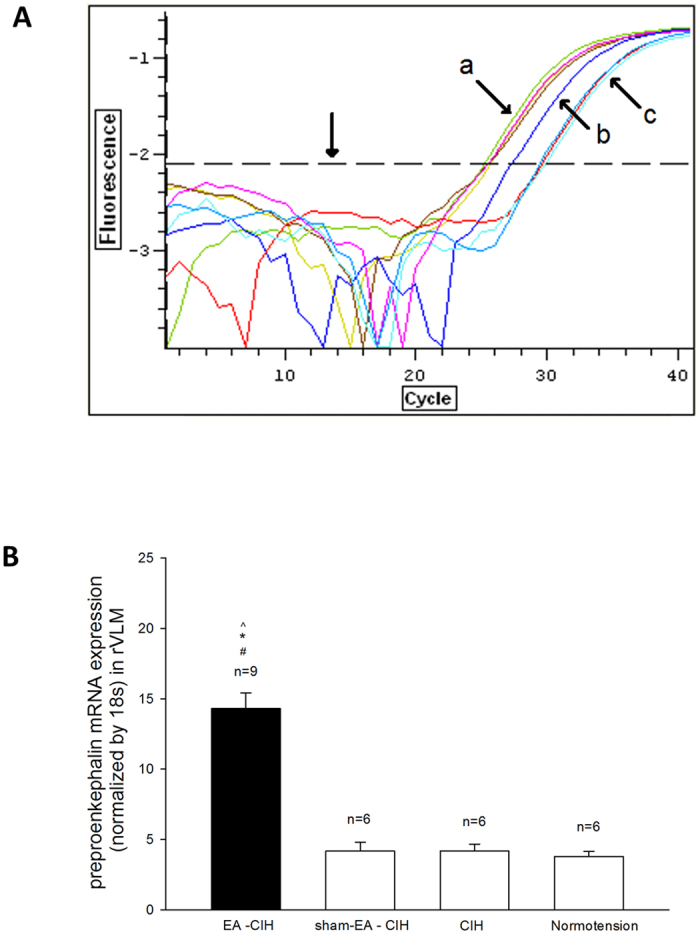
Expression of preproenkephalin (PPE) mRNA in the rVLM of rats. Panel A: Real time PCR traces display levels of fluorescence in a logarithmic scale from four rats, one from each group. The trace crosses the arbitrary line of threshold shown as a dashed horizontal line indicated by the perpendicular arrow. The tissue sample obtained from the EA-treated rat with cold-induced hypertension (CIH) expressed a higher concentration (i.e., at lower number of cycles) of PPE mRNA, represented by the blue curve indicated by (b), compared to control curves (c), which included CIH treated with sham-EA, CIH and normotension (red, blue and light blue curves, respectively). Expression of housekeeping gene 18s (arrow a) was similar among the four groups. They had very similar cycle thresholds. Panel B: Group data show that PPE mRNA, 72 hr after termination of EA treatment, was increased relative to controls. ^, * and # indicate significant differences (P < 0.05) compared respectively with CIH treated with sham-EA, CIH and the normotensive control.

**Figure 4:**
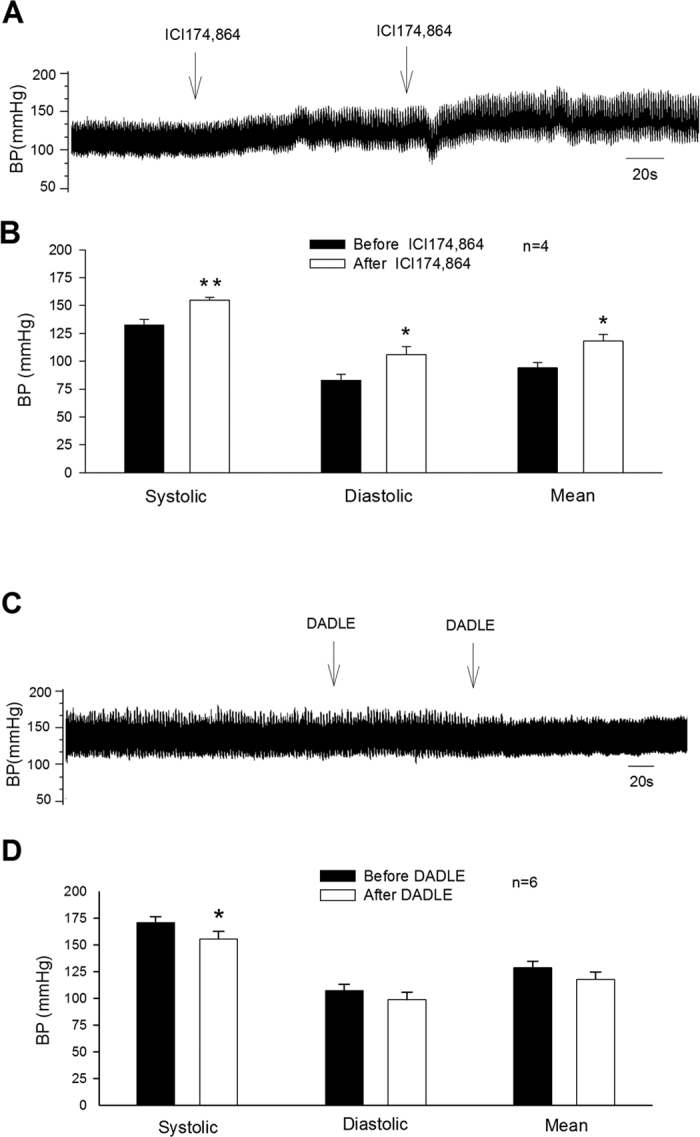
Involvement of rVLM δ-opioid receptors in EA modulation of blood pressure (BP) in rats with cold-induced hypertension (CIH). Panels A,B: δ-opioid receptor blockade in the rVLM reversed EA’s action in lowering BP in CIH rats. Panels C,D: decrease in BP after activation of δ-opioid receptors in the rVLM of CIH rats treated with sham-EA. Panels A,C show original BP tracings before and after bilateral microinjection of a δ-opioid receptor antagonist (ICI 174,864; 1 mM in 50 nl) and agonist (DADLE; 10 nM in 50 nl) into the rVLM. Arrows in Panels A,C indicate sequential bilateral microinjections into the rVLM. In Panels B,D, *indicates significant differences compared to before microinjection, *P < 0.05; **P < 0.01.

**Figure 5:**
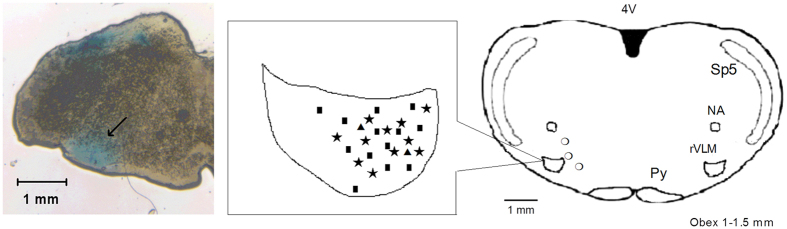
Anatomical locations of microinjection sites in the rat. *Left panel*: An original slide of the medulla oblongata (+1.44 mm from the obex) shows blue-dyed track of a microdialysis probe insertion used for injections. Ventral aspect of blue dye represents site of microinjection in the rVLM, as indicated by arrow. *Middle and right panels*: Composite maps displaying histologically verified sites of microinjections in the rVLM of rats. The *middle panel* displays a magnified area in the *right panel,* indicated by the box. Brain section shows composite of planes of brain stem rostral to the obex (Paxinos and Watson’s atlas). Symbols represent microinjection of ICI 174,864 (★), DADLE (◾), naloxone (▴) and injections outside the rVLM (⚪). All injections were bilateral, although for ease of display, sites are superimposed on the left. rVLM, rostral ventrolateral medulla; Py, pyramidal tract; Sp5, spinal trigeminal nucleus; NA, nucleus ambiguus; 4V, 4th ventricle.

The study conformed to the American Physiological Society’s Guiding Principles for Research Involving Animals and Human Beings. Experimental protocols were approved by the Institutional Animal Care and Use Committee of the University of California at Irvine. A total of 54 male Sprague Dawley rats (150 g) were purchased from Charles River Laboratories International, Inc.). Thirty-six rats were placed into a cold room (6 °C) while 18 rats maintained in room temperature (25 °C) for 11 weeks. They all were provided with standard rodent chow and tap water *ad libitum* and housed in 12-12 hr light-dark cycle. Figure 1 displays the timeline of the experiments.

Blood pressures and heart rates in conscious rats were measured non-invasively with a volume pressure recording sensor and an occlusion tail-cuff (CODA System, Kent Scientific). Digital values were recorded. To evaluate BP, each animal was placed in a restrainer, and the cuff was placed on the tail and inflated and released several times to condition the rat to the procedure. After stabilization, BP was measured five times to acquire an average BP. BPs and HRs in all rats were evaluated weekly (Fig. 1).

### Experimental protocols

All rats were gently restrained in slings for 30 min twice weekly for six weeks to accommodate them to manipulation and to prevent stress associated with this procedure. Each rat was wrapped with a cloth around the body, excluding the four limbs. This method allowed manipulation of the limbs and application of EA at ST36-37 acupoints both safely and effectively in the immobilized conscious rats. The ST36-37 acupoints (overlying deep peroneal nerve below the knee on the hind limb) are analogous to those in humans^53^. Acupuncture needles (0.16 mm diameter) were inserted into the ST36-37 acupoints briefly (~10 s) to allow them to become accustomed to handling and the acupuncture procedure. The rat maintained at room temperature was handled without needle insertion.

After six weeks the rats housed in cold room demonstrated elevated SBP compared to rats maintained at room temperature. The CIH rats were divided randomly into EA, sham-EA or untreated hypertension groups.

#### EA-treated group

Acupuncture needles were inserted bilaterally in ST36-37 and stimulated with a constant current stimulator connected to a stimulus isolation unit (Grass, model S88) to provide EA (2 Hz, 0.1 – 0.4 mA, 0.5 ms duration) for 30 min^21^,^24^,^42^, twice weekly for five weeks (Fig. 1). Correct positioning of the needles at ST36-37 was confirmed by observing slight repetitive paw twitches, indicating stimulation of motor fibers in the mixed nerve bundle comprising the deep peroneal nerve^44^. Of note, motor nerve stimulation does not participate in the EA-cardiovascular response^54^.

#### Sham-EA group

The CIH rats were treated the same as that in the EA group with the exception that needles were inserted but not electrically stimulated.

#### Hypertensive control group

These animals were restrained 30 min twice a week but were not subjected to insertion of acupuncture needles (Fig. 1).

#### Normotensive control group

Rats were maintained at room temperature for 11 weeks with 30 min restraint twice weekly (Fig. 1). No acupuncture was applied to this group.

## Expression of preproenkephalin mRNA in the rVLM

### Tissue collection from rVLM

The rVLM tissue was collected 72 hr following five-weeks of treatment with EA (n = 9) and sham-EA (n = 6), as well as from six hypertensive and six normotensive rats (Fig. 1). All rats were anesthetized with ketamine/xylazine (75–100/5–10 mg/kg, i.p.), then decapitated. Brain tissue was removed and stored in RNA Later solution (Ambion). The rVLM tissue was obtained by positioning a 20-gauge needle stub 0.5 mm lateral to the lateral edge of the pyramid tract next to the caudal edge of the trapezoid body from ventral surface of the brain stem^24^,^42^. The needle stub was inserted perpendicularly 0.5 mm towards dorsally. The first 0.2 mm of harvested tissue from the ventral surface was excluded because it was located outside the rVLM, and the remaining tissue was confirmed to be in the rVLM^22^,^24^,^42^. Bilateral rVLM tissue samples were collected from each rat.

### RNA isolation and real time PCR

Tissue samples were homogenized in a glass tissue grinder (DUALL 20, Kontes Glass Co.) using 800 μl Trizol reagent (Life Technologies). RNA was dissolved in 10 μl nuclease-free water, and the concentration and purity of RNA was determined spectrophotometerically using a Nanodrop ND-1000 spectrophotometer. One hundred ng of total RNA were transcribed using SuperScript II RT and a mixture of oligo (dT) (100 ng/reaction) and random primers (200 ng/reaction; all from Invitrogen). Real-time quantitative PCR was performed with an Opticon 4 using the SYBR green real-time master mix (Bio-Rad). The following primer sequences were used: preproenkephalin, forward (5′-tgc ctt ctt tca aaa tct gg-3′), reverse (5′-ggg gta aag ctc atc cat ct-3′); 18s, forward (5′-cgg aca gga ttg aca gat tg-3′), reverse (5′-acg cca ctt gtc cct cta ag-3′). The sizes of the PCR products for preproenkephalin was 191 bp and for the 18s housekeeping gene 185 bp. The PCR program was 95 °C 10 min, (95 °C 10 s, 59 °C 45 s) x 50 cycles followed by the melting curve analysis (55–90 °C) to verify product specificity. For each sample, the copy number of both preproenkephalin and the 18s housekeeping gene were extrapolated from their respective standard curves. The value of preproenkephalin mRNA expression was normalized by the number of 18s copies and expressed in arbitrary units. Reproducibility of results was determined by performing triplicate measurements of each cDNA aliquot.

## Role of rVLM δ-opioid receptors in EA-modulated hypertension

### Drugs

Naloxone (1 mM) was purchased from Sigma-Aldrich. ICI 174,864 (1 mM)^55^ and DADLE (10 nM)^56^ were δ-opioid receptor antagonist and agonist, respectively. Both of them were purchased from Tocris. ICI 174,864 (5 mg) was dissolved in 100 μl DMSO and then diluted to a concentration of 1 mM with 0.9% normal saline. Naloxone and DADLE were dissolved in the normal saline.

### Surgical preparation

Anesthesia was induced with ketamine (100 mg/kg, i.m.) and α-chloralose (50–60 mg/kg, i.v.). Additional doses of α-chloralose (25–30 mg/kg, i.v.) were administered to maintain an adequate level of anesthesia. A femoral vein and artery were cannulated for administration of fluids and monitoring BP, respectively. HR was derived from the pulsatile BP signal. The trachea was intubated to provide artificial ventilation using a respirator (model 661, Harvard Apparatus). Arterial blood gases were measured with a blood gas analyzer (ABL5, Radiometer America) and maintained within the normal physiological range (PO_2_ >100 mmHg, PCO_2_ 30–40 mmHg and arterial pH 7.35–7.4). Body temperature was maintained between 36 and 38 °C with a heating pad.

### Microinjections in the rVLM

Animals were placed in a stereotaxic head frame. A partial craniotomy was performed to expose the dorsal medulla. Two CMA microdialysis probes (14 mm long with 0.24 mm of tip diameter) used for microinjections were modified by removing microdialysis membrane^43^. They were inserted bilaterally into the rVLM, 1.8–2.3 mm lateral from the midline, 1–1.5 mm rostral to the obex, and advanced 3.0–3.3 mm^21^,^22^,^57^. The probes were connected to 25 μl Hamilton syringes fitted in UMP3 microsyringe injectors (WPI). The microinjections were conducted sequentially into bilateral rVLM.

### Opioid antagonism in EA CIH rats

The naloxone (n = 2) or ICI 174,864 (n = 4; 1 mM in 50 nl) were bilaterally microinjected into the rVLM of CIH rats subjected to eleven repeated applications of EA. This dose of ICI 174,864 in the rVLM attenuates EA modulation of the BP response^19^. Vehicle controls were examined in four rats.

### Delta-opioid receptor stimulation in sham-EA CIH rats

The BP response to rVLM stimulation with DADLE (10 nM in 50 nl) was examined in six sham-EA rats. This dose of DADLE injected into the rVLM markedly lowers BP in acute and sustained hypertension^19^,^46^. In four of these rats, the normal saline was also microinjected into the rVLM two hrs either before or after administration of DADLE.

### Activation and inhibition of delta-opioid receptor in normotensive rats

DADLE or ICI 174,864 was microinjected bilaterally into the rVLM in six normotensive rats.

### Histological confirmation of microinjection sites

We injected 5% Chicago Sky Blue dye (100 nl) at the end of each experiment to identify probe placement. After euthanasia, the medulla oblongata was removed and submerged in 4% paraformaldehyde. Coronal sections (40 μm) were cut with a cryostat microtome (Leica). Using the rat’s atlas^22^ as a guide, sites of microinjections in the medulla were plotted with Corel Presentation software on reconstructed coronal sections^24^,^43^.

## Statistical analysis

Data are presented as means ± SEM. A one-way repeated measure of ANOVA followed post hoc by the Student-Newman Keuls test was used to compare changes in BPs and HRs over time as well as before and after drug microinjection in each group. The difference of BP and HR between two groups was compared using a two-way repeated measure ANOVA followed the Holm-Sidak t-test. Comparisons of preproenkephalin mRNA level between two groups were analyzed using the Student’s *t*-test. Statistical analyses were performed with Sigma Plot (Jandel Scientific). The 0.05 probability level was used to detect significant differences.

## Additional Information

**How to cite this article**: Li, M. *et al*. Repetitive Electroacupuncture Attenuates Cold-Induced Hypertension through Enkephalin in the Rostral Ventral Lateral Medulla. *Sci. Rep.* **6**, 35791; doi: 10.1038/srep35791 (2016).

## References

[b1] Kearney P. M. . Global burden of hypertension: analysis of worldwide data. Lancet 365, 217–223 (2005). 15652604 10.1016/S0140-6736(05)17741-1

[b2] Chobanian A. V. . Seventh report of the Joint National Committee on Prevention, Detection, Evaluation, and Treatment of High Blood Pressure. Hypertension 42, 1206–1252 (2003). 14656957 10.1161/01.HYP.0000107251.49515.c2

[b3] Macklin E. A. . Stop Hypertension with the Acupuncture Research Program (SHARP): results of a randomized, controlled clinical trial. Hypertension 48, 838–845 (2006). 17015784 10.1161/01.HYP.0000241090.28070.4c

[b4] Yin C. . Acupuncture, a promising adjunctive therapy for essential hypertension: a double-blind, randomized, controlled trial. Neurol Res 29 Suppl 1, S98–103 (2007). 17359649 10.1179/016164107X172220

[b5] Brook R. D. . Beyond medications and diet: alternative approaches to lowering blood pressure: a scientific statement from the american heart association. Hypertension 61, 1360–1383 (2013). 23608661 10.1161/HYP.0b013e318293645f

[b6] Longhurst J. C. & Tjen-A-Looi S. C. Acupuncture regulation of blood pressure: two decades of research. Int. Rev. Neurobiol. 111, 257–271 (2013). 24215927 10.1016/B978-0-12-411545-3.00013-4

[b7] Li P. . Long-lasting reduction of blood pressure by electroacupuncture in patients with hypertension: randomized controlled trial. Medical Acupuncture 27, 253–266 (2015). 26392838 10.1089/acu.2015.1106 PMC4555646

[b8] Carlsson C. P. & Sjolund B. H. Acupuncture for chronic low back pain: a randomized placebo-controlled study with long-term follow-up. Clin J Pain 17, 296–305 (2001). 11783809 10.1097/00002508-200112000-00003

[b9] Guerra de Hoyos J. A. . Randomised trial of long term effect of acupuncture for shoulder pain. Pain 112, 289–298 (2004). 15561384 10.1016/j.pain.2004.08.030

[b10] Hata T. . The seasonal variation of blood pressure in patients with essential hypertension. Clin Exp Hypertens A 4, 341–354 (1982). 7074993 10.3109/10641968209060747

[b11] Cold exposure and winter mortality from ischaemic heart disease, cerebrovascular disease, respiratory disease, and all causes in warm and cold regions of Europe. The Eurowinter Group. Lancet 349, 1341–1346 (1997). 9149695

[b12] Yang L. . Outdoor temperature, blood pressure, and cardiovascular disease mortality among 23 000 individuals with diagnosed cardiovascular diseases from China. Eur. Heart J. 36, 1178–1185 (2015). 25690792 10.1093/eurheartj/ehv023 PMC4430682

[b13] Fregly M. J., Kikta D. C., Threatte R. M., Torres J. L. & Barney C. C. Development of hypertension in rats during chronic exposure to cold. J Appl. Physiol 66, 741–749 (1989). 2708203 10.1152/jappl.1989.66.2.741

[b14] Papanek P. E., Wood C. E. & Fregly M. J. Role of the sympathetic nervous system in cold-induced hypertension in rats. J Appl Physiol 71, 300–306 (1991). 1917754 10.1152/jappl.1991.71.1.300

[b15] Maickel R. P., Matussek N., Stern D. N. & Brodie B. B. The sympathetic nervous system as a homeostatic mechanism. I. Absolute need for sympathetic nervous function in body temperature maintenance of cold-exposed rats. J Pharmacol Exp Ther 157, 103–110 (1967). 4382097

[b16] Sun Z. J. & Zhang Z. E. Historic perspectives and recent advances in major animal models of hypertension. Acta Pharmacol. Sin. 26, 295–301 (2005). 15715924 10.1111/j.1745-7254.2005.00054.x

[b17] Kumagai H. . Importance of rostral ventrolateral medulla neurons in determining efferent sympathetic nerve activity and blood pressure. Hypertens. Res. 35, 132–141 (2012). 22170390 10.1038/hr.2011.208 PMC3273996

[b18] Guyenet P. G. The sympathetic control of blood pressure. Nat. Rev. Neurosci. 7, 335–346 (2006). 16760914 10.1038/nrn1902

[b19] Li P., Tjen-A-Looi S. C. & Longhurst J. C. Rostral ventrolateral medullary opioid receptor subtypes in the inhibitory effect of electroacupuncture on reflex autonomic response in cats. Autonomic Neuroscience 89, 38–47 (2001). 11474645 10.1016/S1566-0702(01)00247-8

[b20] Tjen-A-Looi S. C., Li P. & Longhurst J. C. Prolonged inhibition of rostral ventral lateral medullary premotor sympathetic neuron by electroacupuncture in cats. Auton Neurosci.: Basic and Clinical 106, 119–131 (2003). 10.1016/S1566-0702(03)00076-6 12878081

[b21] Zhou W., Tjen-A-Looi S. & Longhurst J. C. Brain stem mechanisms underlying acupuncture modality-related modulation of cardiovascular responses in rats. J. Appl. Physiol. 99, 851–860 (2005). 15817715 10.1152/japplphysiol.01365.2004

[b22] Paxinos G. & Watson C. The rat brain in stereotaxic coordinates. Academic Press (2009). 10.1016/0165-0270(80)90021-7 6110810

[b23] Tjen-A-Looi S. C., Li P. & Longhurst J. C. Role of medullary GABA, opioids, and nociceptin in prolonged inhibition of cardiovascular sympathoexcitatory reflexes during electroacupuncture in cats. Am J Physiol 293, H3627–H3635 (2007). 10.1152/ajpheart.00842.2007 17890425

[b24] Li M., Tjen-A-Looi S. C., Guo Z. L. & Longhurst J. C. Repetitive electroacupuncture causes prolonged increased met-enkephalin expression in the rVLM of conscious rats. Auton. Neurosci 170, 30–35 (2012). 22841685 10.1016/j.autneu.2012.07.001 PMC3461830

[b25] Judy W. V. & Farrell S. K. Arterial baroreceptor reflex control of sympathetic nerve activity in the spontaneously hypertensive rat. Hypertension 1, 605–614 (1979). 541053 10.1161/01.hyp.1.6.605

[b26] Nakamura Y. . Central attenuation of aortic baroreceptor reflex in prehypertensive DOCA-salt-loaded rats. Hypertension 12, 259–266 (1988). 3169941 10.1161/01.hyp.12.3.259

[b27] Guo G. B., Thames M. D. & Abboud F. M. Arterial baroreflexes in renal hypertensive rabbits. Selectivity and redundancy of baroreceptor influence on heart rate, vascular resistance, and lumbar sympathetic nerve activity. Circ Res 53, 223–234 (1983). 6883646 10.1161/01.res.53.2.223

[b28] Wang X. . AAV-based RNAi silencing of NADPH oxidase gp91(phox) attenuates cold-induced cardiovascular dysfunction. Hum Gene Ther 23, 1016–1026 (2012). 22888847 10.1089/hum.2012.078 PMC3440021

[b29] Brennan P. J., Greenberg G., Miall W. E. & Thompson S. G. Seasonal variation in arterial blood pressure. Br Med J (Clin Res Ed) 285, 919–923 (1982). 10.1136/bmj.285.6346.919 PMC1499985 6811068

[b30] Downs N. M., Kirk K. & MacSween A. The effect of real and sham acupuncture on thermal sensation and thermal pain thresholds. Arch. Phys. Med. Rehabil. 86, 1252–1257 (2005). 15954068 10.1016/j.apmr.2004.10.037

[b31] Amand M., Nguyen-Huu F. & Balestra C. Acupuncture effect on thermal tolerance and electrical pain threshold: a randomised controlled trial. Acupunct. Med. 29, 47–50 (2011). 21139035 10.1136/aim.2010.002485

[b32] Lundeberg T., Eriksson S., Lundeberg S. & Thomas M. Acupuncture and sensory thresholds. Am. J. Chin Med. 17, 99–110 (1989). 2534464 10.1142/S0192415X89000176

[b33] Leung A., Khadivi B., Duann J. R., Cho Z. H. & Yaksh T. The effect of Ting point (tendinomuscular meridians) electroacupuncture on thermal pain: a model for studying the neuronal mechanism of acupuncture analgesia. J. Altern. Complement Med. 11, 653–661 (2005). 16131289 10.1089/acm.2005.11.653

[b34] Wang S. M., Lin E. C., Maranets I. & Kain Z. N. The impact of asynchronous electroacupuncture stimulation duration on cold thermal pain threshold. Anesth. Analg. 109, 932–935 (2009). 19690269 10.1213/ane.0b013e3181ad9292

[b35] Stux G. & Pomeranz B. Basics of acupuncture. Springer-Verlag, Berlin (1998).

[b36] Zhou W., Fu L.-W., Tjen-A-Looi S. C., Li P. & Longhurst J. C. Afferent mechanisms underlying stimulation modality-related modulation of acupuncture-related cardiovascular responses. J Appl Physiol 98, 872–880 (2005). 15531558 10.1152/japplphysiol.01079.2004

[b37] Tjen-A-Looi S. C., Li P. & Longhurst J. C. Medullary substrate and differential cardiovascular response during stimulation of specific acupoints. Am J Physiol 287, R852–R862 (2004). 10.1152/ajpregu.00262.2004 15217791

[b38] Rapsomaniki E. . Blood pressure and incidence of twelve cardiovascular diseases: lifetime risks, healthy life-years lost, and age-specific associations in 1.25 million people. Lancet 383, 1899–1911 (2014). 24881994 10.1016/S0140-6736(14)60685-1 PMC4042017

[b39] Guo Z.-L., Moazzami A. R. & Longhurst J. C. Electroacupuncture induces c-Fos expression in the rostral ventrolateral medulla and periaqueductal gray in cats: relation to opioid containing neurons. Brain Res. 1030, 103–115 (2004). 15567342 10.1016/j.brainres.2004.09.059

[b40] Li P., Tjen-A-Looi S. C., Guo Z. L., Fu L.-W. & Longhurst J. C. Long-loop pathways in cardiovascular electroacupuncture responses. J. Appl. Physiol. 106, 620–630 (2009). 19074569 10.1152/japplphysiol.91277.2008 PMC2644252

[b41] Yao T., Andersson S. & Thoren P. Long-lasting cardiovascular depression induced by acupuncture-like stimulation of the sciatic nerve in unanaesthetized spontaneously hypertensive rats. Brain Res. 240, 77–85 (1982). 7201339 10.1016/0006-8993(82)90645-x

[b42] Li M., Tjen-A-Looi S. C. & Longhurst J. C. Electroacupuncture enhances preproenkephalin mRNA expression in rostral ventrolateral medulla of rats. Neurosci Lett 477, 61–65 (2010). 20399834 10.1016/j.neulet.2010.04.025 PMC2892533

[b43] Li M., Tjen-A-Looi S. C., Guo Z. L. & Longhurst J. C. Electroacupuncture modulation of reflex hypertension in rats: role of cholecystokinin octapeptide. Am J Physiol 305, R404–R413 (2013). 10.1152/ajpregu.00196.2013 PMC3833400 23785073

[b44] Chao D. M. . Naloxone reverses inhibitory effect of electroacupuncture on sympathetic cardiovascular reflex responses. Am. J. Physiol. 276, H2127–H2134 (1999). 10362696 10.1152/ajpheart.1999.276.6.H2127

[b45] Zamir N., Simantov R. & Segal M. Pain sensitivity and opioid activity in genetically and experimentally hypertensive rats. Brain Res 184, 299–310 (1980). 6243503 10.1016/0006-8993(80)90800-8

[b46] Sun S. Y., Liu Z., Li P. & Ingenito A. J. Central effects of opioid agonists and naloxone on blood pressure and heart rate in normotensive and hypertensive rats. Gen Pharmacol 27, 1187–1194 (1996). 8981066 10.1016/s0306-3623(96)00055-9

[b47] Li P., Zhu D. N., Kao K. M., Lin Q. & Sun S. Y. Role of acetylcholine, corticoids and opioids in the rostral ventrolateral medulla in stress-induced hypertensive rats. Biol Signals 4, 124–132 (1995). 8750938 10.1159/000109432

[b48] Cox R. H. Influence of chloralose anesthesia on cardiovascular function in trained dogs. Am J Physiol 223, 660–667 (1972). 5055323 10.1152/ajplegacy.1972.223.3.660

[b49] Shebetai R., Fowler N. & HURLBURT O. Hemodynamics studies of dogs under pentobarbitol and morphine chloralose anesthesia. J Surg Res 3, 263–267 (1963). 14043083 10.1016/s0022-4804(63)80053-0

[b50] Priano L. L., Traber D. L. & Wilson R. D. Barbiturate anesthesia: an abnormal physiologic stimulation. J Pharmacol Exp Ther 165, 126–135 (1969). 5782829

[b51] Sander G. E., Lowe R. F. & Giles T. D. The effects of barbiturates upon the hemodynamic responses to intravenous methionine-enkephalin in dogs: modulation by the GABA complex. Peptides 7, 259–265 (1986). 3016681 10.1016/0196-9781(86)90223-8

[b52] Kasamatsu K. & Sapru H. N. Attenuation of aortic baroreflex responses by microinjections of endomorphin-2 into the rostral ventrolateral medullary pressor area of the rat. Am J Physiol Regul Integr Comp Physiol 289, R59–R67 (2005). 15718394 10.1152/ajpregu.00007.2005

[b53] Hua X. B. Acupuncture manual for small animals experimental acupuncture. Shanghai Science and Technology Publisher, Shanghai (1994).

[b54] Li P., Tjen-A-Looi S. C. & Longhurst J. C. Excitatory projections from arcuate nucleus to ventrolateral periaqueductal gray in electroacupuncture inhibition of cardiovascular reflexes. Am. J. Physiol. 209, H2535–H2542 (2006). 10.1152/ajpheart.00972.2005 16399864

[b55] Cotton R., Giles M. G., Miller L., Shaw J. S. & Timms D. ICI 174864: a highly selective antagonist for the opioid delta-receptor. Eur J Pharmacol 97, 331–332 (1984). 6323195 10.1016/0014-2999(84)90470-9

[b56] Suh H. H. & Tseng L. F. Different types of opioid receptors mediating analgesia induced by morphine, DAMGO, DPDPE, DADLE and beta-endorphin in mice. Naunyn Schmiedebergs Arch Pharmacol 342, 67–71 (1990). 1976234 10.1007/BF00178974

[b57] Tjen-A-Looi S. C., Guo Z. L., Li M. & Longhurst J. C. Medullary GABAergic mechanisms contribute to electroacupuncture modulation of cardiovascular depressor responses during gastric distention in rats. Am. J. Physiol. 304, R321–R332 (2013). 10.1152/ajpregu.00451.2012 PMC3602723 23302958

